# Human acellular amniotic membrane implantation for lower third nasal reconstruction: a promising therapy to promote wound healing

**DOI:** 10.1186/s41038-018-0136-x

**Published:** 2018-12-18

**Authors:** Si-Liang Xue, Kai Liu, Ornella Parolini, Yue Wang, Li Deng, Yong-Can Huang

**Affiliations:** 1 Department of Dermatology and Venereology, West China Hospital, Sichuan University, Chengdu, Sichuan China; 2 Chengdu Qingshan Likang Pharmaceutical Co., Ltd., Chengdu, People’s Republic of China; 3 Centro di Ricerca E. Menni, Fondazione Poliambulanza-Istituto Ospedaliero, Brescia, Italy; 4 Istituto di Anatomia Umana e Biologia Cellulare, Università Cattolica del Sacro Cuore Facoltà di Medicina e Chirurgia, Rome, Italy; 5 Department of Ultrasound, Peking University Shenzhen Hospital, Shenzhen, People’s Republic of China; 6 Laboratory of Stem Cell and Tissue Engineering, State Key laboratory of Biotherapy, West China Hospital, Sichuan University, Chengdu, Sichuan China; 7 Shenzhen Engineering Laboratory of Orthopaedic Regenerative Technologies, Orthopaedic Research Center, Peking University Shenzhen Hospital, Shenzhen, China; 8 Shenzhen Key Laboratory of Spine Surgery, Department of Spine Surgery, Peking University Shenzhen Hospital, 1120 Lianhua Road, Futian District, Shenzhen, 518036 People’s Republic of China

**Keywords:** Acellular amniotic membrane, Nasal reconstruction, Wound healing, Repair

## Abstract

**Background:**

The lower third of the nose is one of the most important cosmetic units of the face, and its reconstructive techniques remain a big challenge. As an alternative approach to repair or regenerate the nasal tissue, the biomaterial-based strategy has been extensively investigated. The aim of this study is to determine the safety and efficacy of human acellular amniotic membrane (HAAM) to repair the full-thickness defects in the lower third of the nose in humans.

**Methods:**

In this study, 180 patients who underwent excision of skin lesions of the lower third of the nose from 2012 to 2016 were included; of the patients, 92 received HAAM and Vaseline gauze treatments, and the other 88 patients received Vaseline gauze treatment only. The haemostasis time and the duration of operation were recorded during surgery; after surgery, the time to pain disappearance, scab formation and wound healing, and the wound healing rate were measured.

**Results:**

Immediately after the HAAM implantation, a reduction of the haemostasis time and an accelerated disappearance of pain were observed. Compared with the control group, the formation and detachment of scab in patients who received the HAAM implantation were notably accelerated, postoperatively. When the diameter of the lesion exceeded 5 mm, the HAAM implantation was found to enhance the wound healing, although this enhancement was not seen when the diameter was less than 5 mm. Additionally, the HAAM implantation significantly reduced bleeding, wound infection and scar formation, postoperatively.

**Conclusions:**

HAAM-assisted healing is a promising therapy for lower third nasal reconstruction leading to rapid wound healing and fewer complications and thus has considerable potential for extensive clinical application in repairing skin wounds.

**Trial registration:**

ChiCTR1800017618, retrospectively registered on July 08, 2018.

## Background

The lower third of the nose is composed of the bilateral ala and soft triangles, the central tip and columella; its topography is characterised by interwoven concavities, convexities and varying skin thickness [1]. As one of the most important cosmetic units of the face, the lower third nasal reconstruction remains a big challenge for reconstructive surgeons and researchers. The ideal reconstruction should restore the normal anatomy while limiting the flaws to the nasal cosmetic unit. To achieve this goal, a myriad of reconstructive strategies have been developed that include numerous flaps and skin grafting [1–3].However, interest in developing alternative approaches of reconstruction—particularly biomaterial-based methods that repair or regenerate the nasal tissue—is growing [4, 5].

Human amniotic membrane (AM) has been long used as an attractive biomaterial for skin defect healing. Taken from the innermost layer of the placenta, the human AM is highly abundant and readily available without invasive harvesting and ethical concerns. Several decades ago, the fresh and fresh-frozen human AM were used to dress partial-thickness burns in humans, offering substantial pain relief [6, 7]. Human AM also prevents infection and accelerates healing in the treatment of chronic ulcers [8, 9], necrotising fasciitis [10], epidermolysis bullosa [11] and other cutaneous diseases [12]. Similar outcomes were determined after the human AM was dehydrated or freeze-dried [13–16].

Decellularised AM was developed to prevent sensitisation and immune reactions, minimise potential infection and reduce the high costs related to the application of living cellularised graft [17]. While human acellular AM (HAAM) provides a nearly unlimited source for skin defect healing, it is unknown whether HAAM possesses the potential to repair nasal tissue in humans. After the procedure of decellularisation and dehydration was defined [18], the three-dimensional HAAM was found to be capable of supporting the attachment and proliferation of allogenic cells [19], it was further determined that HAAM increased the epidermal thickness and improved epidermal healing in mice [20, 21], rats [22] and pigs [23]. These achievements accelerated the clinical application of HAAM for nasal skin defects reconstruction.

While the lower third of the nose is critical for the aesthetic appearance of the face, this region is commonly affected by unintended and deliberate injuries. This clinical study therefore investigates the safety and feasibility of implanting HAAM to repair successfully the full-thickness defects in the lower third of the nose.

## Methods

### Preparation of HAAM

The HAAM was provided by Chengdu Qingshan Likang Pharmaceutical Co., Ltd., which has been approved by the China Food and Drug Administration. The manufacture process (Chinese patent no. ZL200410036792.4) was conducted according to the Good Manufacturing Practice in People’s Republic of China. In brief, the HAAM was separated from the placentas of consenting mothers and rinsed with sterile distilled water to remove the residual blood. Serological examination was performed to exclude the possibility of human immunodeficiency virus type I, syphilis and human hepatitis virus types B and C. The fresh HAAM was submerged in a solution of methanol and chloroform, rinsed with sterile distilled water and perfused with sodium dodecyl sulfate (SDS, Sigma) in distilled water for at least 5 h. The decellularisation process was done using 0.25% trypsin for 6 h and was then rinsed with a saline solution. The HAAM was freeze-dried under a vacuum, cut into pieces 1 cm × 1 cm, packed into hermetic packages and sterilised with gamma radiation. The HAAM was stored aseptically at room temperature until implantation.

### Histology and scanning electron microscope (SEM) analysis

The HAAM cut into sizes of 0.5 × 0.5 cm was stained directly with hematoxylin and eosin (H&E); also, it was embedded in paraffin wax and cut into 5-μm-thick sections for H&E staining for cross-section observation. In addition, the HAAM was subjected to critical point drying and the ultrastructure was observed using a SEM.

### Patients

From July 2012 to August 2016, 180 patients from West China Hospital (Chengdu, Sichuan, People’s Republic of China) who underwent excisions of skin lesions of the lower third of the nose and secondary healing were included in this study. The inclusion criteria were (1) patients who had directly excisable lesions at the nasal tip or ala nasi regions and underwent secondary healing, (2) no abnormality during preoperative examination and (3) no infection at the surgical areas. The exclusion criteria were (1) patients aged younger than 12 years, (2) patients whose defects were unsuitable for secondary healing and (3) patients who had a long-term history of chronic diseases, such as diabetes, renal failure, or immunosuppression. All patients were informed of the full content of the clinical trial and signed the consent forms preoperatively. This trial has been registered in Chinese Clinical Trial Registry with the registration number ChiCTR1800017618. This study was approved by Ethical Committee of West China Hospital, Sichuan University.

### Groups and surgical procedures

The surgeries were performed by the same team at the Department of Dermatology and Venereology, West China Hospital, Sichuan University. The included patients were randomly assigned to either the HAAM treatment group or the control group (Vaseline gauze treatment) by the randomisation serial numbers from the statistical program SPSS software (SPSS 19.0; IBM., Armonk, NY, USA). The following details of all patients were recorded: gender, age, location of skin lesions, size of skin defects after skin lesion excision, postoperative complications (bleeding, infection, hematoma and appearance impairment) and recurrence rate of skin lesions. Before surgery, each patient received a local anaesthesia with 0.1% lidocaine in the supine position; the skin lesion was excised using a no. 15 blade to reach the subcutaneous tissues, and the compression haemostasis was conducted using the wet gauze. In the treatment group, the HAAM was cut into small pieces (2 × 2 mm) and placed into the bottom and wall of the defects; the wound was then covered with Vaseline gauze and dressed using the sterile gauze. In the control group, the wound was directly covered with Vaseline gauze and dressed using the sterile gauze. The gauze was replaced the next day postoperatively, and changed every 2 days until a scab formed. After the scab detached, the patients returned to the hospital for clinical assessments.

### Clinical assessments

During surgery, the haemostasis time and the duration of operation were recorded. Haemostasis time refers to the interval from the removal of the lesion to the time the wound was ready for dressing without bleeding; the duration of operation refers to the interval from the beginning to the end of the surgery when bandaging was complete. After surgery, the time to pain disappearance, scab formation and wound healing, and the wound healing rate were measured. Time to pain disappearance is the interval from when the anaesthetic effect ended and pain was noticeable after surgery to when the pain disappeared; time to scab formation is the interval from the end of surgery to the drying of the wound surface with the formation of a scab; wound healing time is the interval from the scab formation to when the scab completely detaches; and the wound healing rate is the difference between the maximum diameter of the surgical wound and the maximum diameter of the surgical defect at 15 days after surgery. The diameter of the lesion was classified as < 5 mm, 5–10 mm and > 10 mm. At 3 months post operation, the patients were recruited to observe the wound surface.

### Statistical analysis

Statistical analysis was performed using SPSS software (SPSS 19.0; IBM., Armonk, NY, USA). Student’s *t*, chi-square and Fisher’s exact tests were performed to compare the characteristic data of patients and the data of clinical assessments between the two groups. Significance was accepted when *p* value < 0.05.

## Results

### Characteristics of the HAAM

**Fig. 1:**
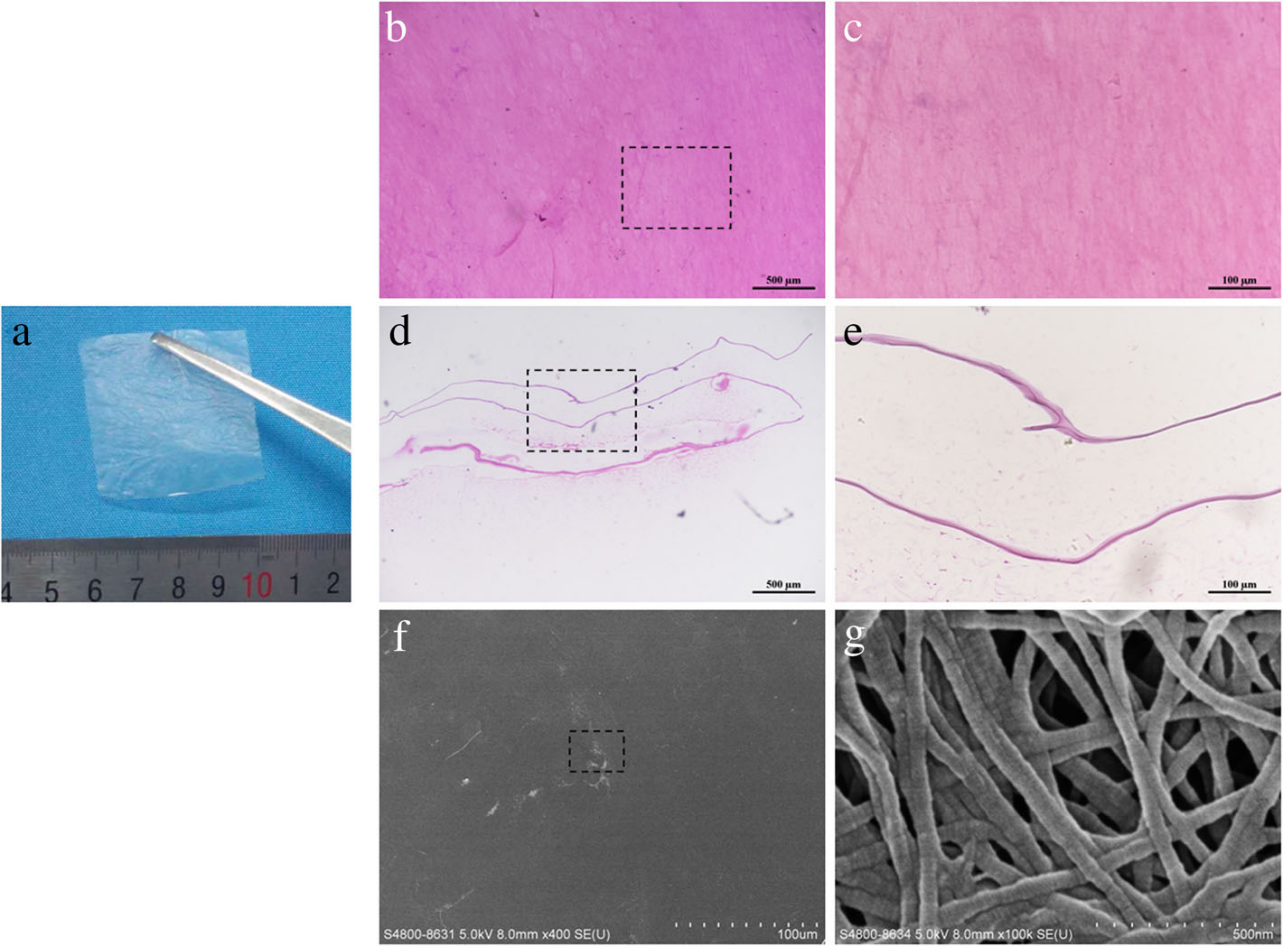
Characteristics of the human acellular amniotic membrane (HAAM). **a** Gross view of the HAAM before implantation. **b**, **c** Images of hematoxylin and eosin (H&E) staining indicate the resident cells were completely removed after the decellularisation procedures. **d**, **e** Cross-sectional morphology of the HAAM after H&E staining. **f**, **g** Scanning electron microscope (SEM) results of the HAAM illustrating the collagen structure

As shown in Fig. 1a, b, c, d, e, the HAAM was a translucent membrane with a thickness of about 10 μm, and no visible cellular materials were evident; the results of the SEM scan indicated the presence of the extracellular matrix components and the collagen scaffold (with the diameter of 80–100 nm) in the HAAM (Fig. 1f, g).

### HAAM implantation enhanced wound healing

**Fig. 2:**
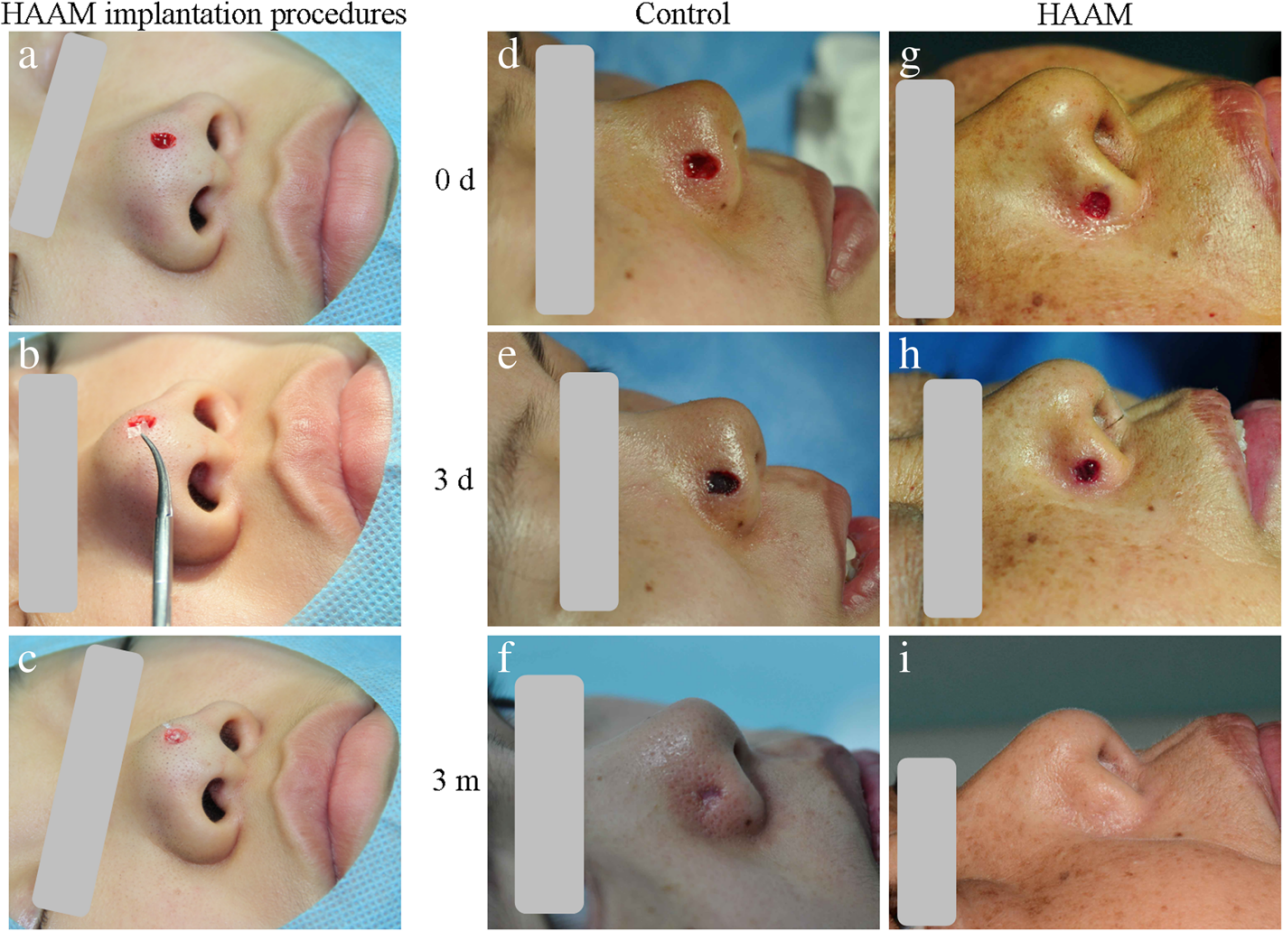
The procedures of human acellular amniotic membrane (HAAM) implantation in the defect of ala nasi and representative cases of the wound healing at the control and the HAAM implantation groups. **a** Excision of the lesion. **b** Cutting the HAAM into small pieces for implantation. **c** Pieces of HAAM were placed into the bottom and wall of the defect. **d-f** Wound healing process of the patient of the control group. **g-i** Immediately after excision, 3 days after HAAM implantation and 3 months after HAAM implantation. **e** More bleeding made thicker scab in wound in the control group, which would hinder the formation of granulation tissue and the creep of epidermis. **h** The scab was much thinner and wetter to form more granulation tissue. **f**, **i** After the scab detached from the wound at 3 months postoperatively, the scar after HAAM treatment was much shallow than that of the control group

In this study, 92 patients (age 32.63 ± 8.64 years) received HAAM treatment and the other 88 patients (age 30.35 ± 8.33 years) received the conventional treatment (Table 1). No significant difference was found between these two groups regarding gender, age and location of skin lesions (*p* > 0.05). The HAAM was cut into small pieces and placed into the bottom and wall of the defects (Fig. 2a, b, c); so the HAAM treatment took much longer time than the treatment given to the control group, with mean values of 22.55 and 20.95 min, respectively.

**Table 1:** Patients data and duration of operations in HAAM group and control group

Variable	HAAM group	Control group	*P* value
Gender (F/M)	53/39	57/31	0.32
Age (year, mean ± SD)	32.63 ± 8.64	30.35 ± 8.33	0.09
Location (*n*)			
Ala nasi	59	47	
Nasal tip	33	41	
Duration of operation (min, mean ± SD)	22.55 ± 3.20	20.95 ± 3.72	< 0.05

*HAAM* human acellular amniotic membrane, *SD* standard deviation

**Fig. 3:**
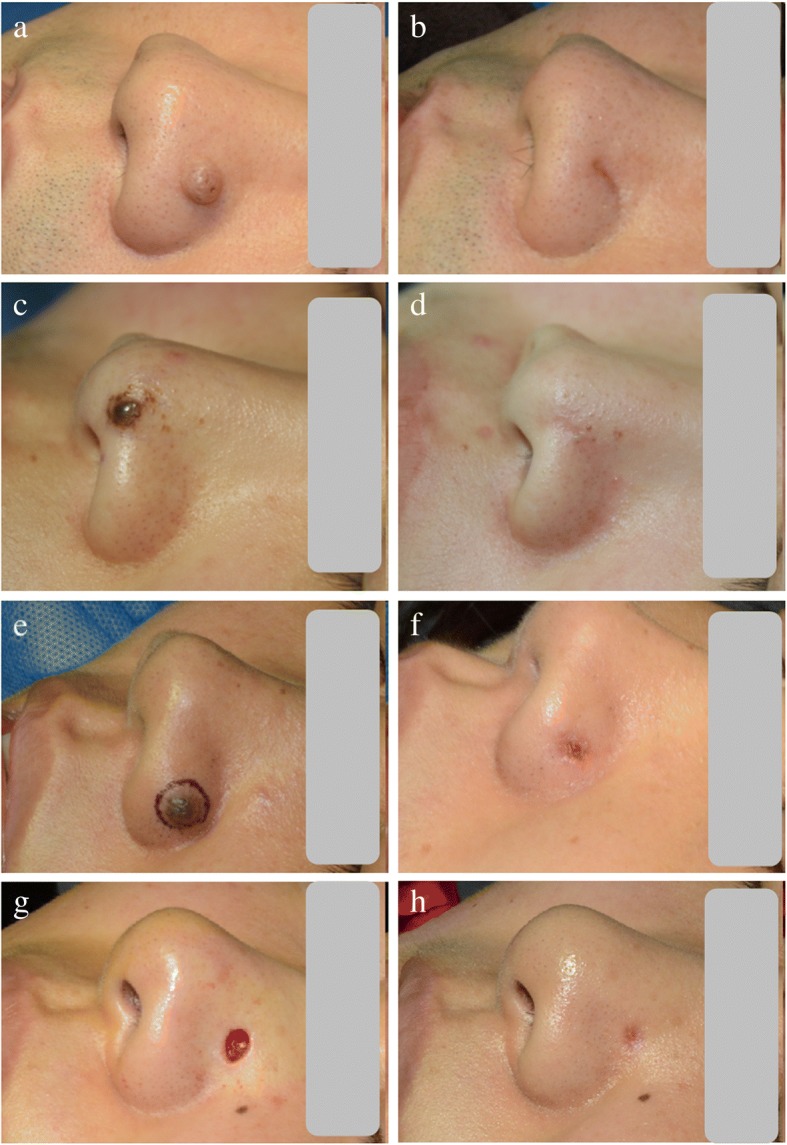
Representative cases of the use of human acellular amniotic membrane (HAAM) for the wound healing at different locations. **a**, **c**, **e**, **g** Before HAAM implantation; **b**, **d**, **f, h** 1 month after HAAM treatment

Several enhancements without adverse events or recurrences of skin lesions were recorded after the HAAM was implanted in the defects of 92 patients and control group (Fig. 2 d, e, f, g, h, i). As shown in Table 2, a significant reduction in the haemostasis time was seen in the HAAM treatment group (*p* < 0.001). Second, the HAAM was found to accelerate the disappearance of pain after surgery, with a mean value of 6.36 h (*p* < 0.001). In addition, when compared with the values of the control group, HAAM implantation was found to noticeably accelerate the formation (4.75 days) and detachment (6.70 days) of scabs postoperatively (*p* <  0.001). After surgery, when the diameter of the lesion exceeded 5 mm, the HAAM was found to enhance the wound healing significantly when compared with the results of the control group (*p* <  0.05); nevertheless, this enhancement was not found when the diameter was less than 5 mm. The HAAM notably reduced the occurrence of bleeding, wound infection and scar formation after surgery (*p* <  0.05), and no puckering or buckling of the healing sites was noted in these patients after the HAAM treatment (Fig. 3a, b, c, d, e, f, g, h).

**Table 2:** Clinical outcomes and postoperative complications after HAAM implantation surgery

Variable	HAAM group	Control group	*P* value
Haemostasis time (min, mean ± SD)	3.11 ± 1.14	5.48 ± 1.53	< 0.001
Disappearance time of pain (hour, mean ± SD)	6.36 ± 1.90	11.11 ± 3.55	< 0.001
Time of scab formation (day, mean ± SD)	4.75 ± 1.13	6.84 ± 1.07	< 0.001
Time of scab detachment (day, mean ± SD)	6.70 ± 0.98	8.72 ± 1.99	< 0.001
Healing rate (mm, mean ± SD)			
0–5 mm	3.54 ± 0.88	3.29 ± 0.69	0.30
5–10 mm	6.79 ± 1.25	5.26 ± 0.97	< 0.001
> 10 mm	10.27 ± 1.62	8.45 ± 0.93	< 0.05
Bleeding (*n*)	2	7	< 0.001
Wound infection (*n*)	4	14	< 0.05
Scar (*n*)	4	12	< 0.05

*HAAM* human acellular amniotic membrane, *SD* standard deviation

## Discussion

To our knowledge, this is one of the primary studies to determine the safety and efficacy of the proof of concept that HAAM augments the reconstruction of lower third nasal in humans. The observed advantages of HAAM implantation include promoting haemostasis, accelerating the wound healing and pain relief and preventing bleeding, infection and scar formation; additionally, HAAM has the benefits of accessibility and ease of use; this new graft-assisted healing thus possesses significant potential for extensive clinical application in repairing skin wounds.

Before implantation, the HAAM was cut into small pieces and placed at the bottom and wall of the defect because large pieces of HAAM could not cover the wound well. After the small pieces of HAAM were randomly placed on the moist defect, they were attached firmly to achieve effective haemostasis and pain relief. After surgery, bleeding induced the formation of a scab, which likely inhibited the formation of the granulation tissue and the creep of the epidermis; finally, a pitting scar formed after the scab detachment [24]. In this study, HAAM implantation reduced the postoperative bleeding, made thinner scab and enhanced wound healing with an improved cosmetic outcome, which may be related to its function of haemostasis and enhancement of the formation of granulation tissue within 24 h. However, surgeons require more surgery time (2–3 min) to cut the HAAM and insert it into the defect piece by piece.

Fresh, cryopreserved or dehydrated AM has been used successfully for wound healing in experimental models and patients for several decades, owing to the properties of reepithelialising, anti-inflammatory and scar prevention without rejection or allergic reaction [7, 25–27]. It is well known that the cells isolated from the AM release immunomodulant factors that control the immune response and induce a decrease in the inflammatory response (Th1 and Th17) [28], an increase of regulatory cells (M2 and Treg) [29], and cytokines, such as basic fibroblast growth factor, platelet-derived growth factor and epidermal growth factor, which facilitate the wound repair and regeneration [30]. Additionally, the dynamic interactions between the cytokines and the extracellular matrix components of AM likely further enhance this regenerative procedure [31]. At the cellular level, AM enhanced the proliferation [32] and migration [33] of keratinocytes through modulating the transforming growth factor-β pathway, and this cellular response is essential for wound healing. A similar regenerative capacity was seen in the decellularised AM as a wound dressing. Without procuring any cytotoxic effects, HAAM supported the attachment and proliferation of dermal fibroblasts, keratinocytes and microvascular endothelial cells [34], and the delivery of stem cells [35]. Recently, using a rat model, it was indicated that HAAM was able to promote wound healing and alleviate wound inflammation and scar formation by enhancing the secretion of vascular endothelial growth factor and alpha-smooth muscle actin [36]. Even though the AM was solubilised, the acceleration of the wound closure and prevention of the wound contraction was recorded in a full-thickness murine wound model [21]. The above findings in animals suggest encouraging outcomes of HAAM for the lower third nasal reconstruction in humans in this study. Although the dehydrated AM has been reported to treat traumatic wounds to the nasal tip in one case [16], our study is the first randomised controlled trial to investigate the feasibility of HAAM in the treatment of circular skin defects for nasal reconstruction and to demonstrate excellent healing with minimal aesthetic impact.

Repairing nasal defects, especially the full-thickness defects, is one of the leading challenges for plastic surgeons. In traditional closure surgery, some healthy skin is removed to form an elliptical defection which is then closed directly; however, this surgery often distorts the tissue surrounding the defect, causing puckering or buckling. Graft-assisted healing strategies provide attractive alternative tools for wound closure, especially the biomaterial-dependent ones [37–40]. While these biomaterial-based approaches can restore the normal anatomy, they also limit the loss of the cosmetic unit. In this study, we pioneered the clinical utilisation of HAAM for nasal reconstruction, which should add value for the translation of biomaterial-based therapies.

This preliminary study has several limitations. More quantitative comparisons of the clinical parameters between these two groups (such as haemostasis and pain) would perfect the experimental design. It is still unknown whether the locations of the wound (ala nasi and nasal tip) affect the healing potential of HAAM. Histological results were not available after the HAAM implantation because it is unethical and unacceptable for the patients to perform a biopsy on healed skin. Additionally, the underlying mechanism of the regenerative capacity of HAAM remains unclear and needs further investigation, even though the studies in the field of fresh or dehydrated AM have provided some clues. It is still unclear whether this HAAM-based approach is suitable for other skin diseases such as burns and diabetic foot ulcers. A reliable and simple method to standardise the preparation of HAAM will be largely required to develop more standardised procedures for its clinical use. A randomised, multicentre, double-blind and controlled trial is needed to further test the safety and efficacy of HAAM implantation.

## Conclusion

In this clinical investigation, HAAM implantation was approved to be feasible and effective for lower third nasal reconstruction. The accelerated wound healing, the reduced postoperative complications, preservation of the aesthetic appearance and the ease of use make the HAAM-based approach a promising procedure for treating skin defects. This new graft-assisted healing has significant potential for extensive use in repairing skin wounds.

## Abbreviations

AM: Amniotic membrane
H&E: Hematoxylin and eosin
HAAM: Human acellular amniotic membrane
SEM: Scanning electron microscope
